# Drug Resistance and Novel Targets for Cancer Therapy: An Overview of Recent Findings

**DOI:** 10.3390/biomedicines12040816

**Published:** 2024-04-08

**Authors:** Gabriel Ouverney, Déborah Hottz, Bruno Kaufmann Robbs

**Affiliations:** 1 Postgraduate Program in Applied Science for Health Products, Faculty of Pharmacy, Fluminense Federal University, Niteroi 24020-141, RJ, Brazil; ouverneygabriel@id.uff.br; 2 Basic Science Department, Health Institute of Nova Friburgo, Fluminense Federal University, Nova Friburgo 28625-650, RJ, Brazil; deborah.hottz@gmail.com

## 1. Introduction

The complex nature of cancer cells poses a challenge to the scientific community. Intrinsic and acquired resistance mechanisms are implied in non-responsive initial cancer treatment or later resistance development, respectively, thus significantly limiting the therapeutic options [1,2].

In this scenario, the discovery of new bioactive molecules, development of techniques, or repurposing of drugs aiming to target diverse cellular components are imperative. This approach is crucial to overcome resistance-related factor such as DNA repair [3], evasion of cell death [4], mutation in targets [5], and the activation of alternative pathways post-oncoprotein inhibition [6]. In this Special Issue, twenty-four works have explored the various facets of cancer resistance mechanisms and novel targets.

## 2. Research Articles

The incorporation of drug combination in chemotherapy represents a significant method in the attempt to overcome resistance. Several works in this Special Issue have demonstrated that the association of two or more drugs can yield important antitumoral effects. The article published by Bhoir et al. (Article 1) studied one of the mechanisms of resistance developed by prostate cancer. They demonstrated that the use of a TLK1 inhibitor, J54, causing the stop in homologous recombination repair, was able to resensitize cisplatin (CPT)-resistant cells. It was observed that RAD54 phosphorylation was reduced when J54 was administered, showing that J54 could efficiently block the TLK1 pathway. Therefore, it is assumed that the combination of CPT and J54 may be a good strategy to overcome the resistance against DNA-damaging drugs. Following a rationale of the combination of molecules to enhance antitumoral efficiency, Mansur et al. (Article 2) tested the synergism between Trastuzumab and the PARP inhibitor Niraparib against HER2+ breast cancer. Even though Trastuzumab treatment does not involve DNA breaking, they observed the better inhibition of cancer cells in combined treatment. Glucose uptake, essential for the viability of cancer cells, was reduced in the combination treatment of Trastuzumab and Niraparib in comparison to therapy alone. Data published by Mao et al., Article 3 in this Special Issue, investigated the effects of arsenic trioxide (ATO) in non-small-cell lung cancer (NSCLC) patients with pleural effusion. Their data suggest that ATO could be an important sensitizer of NSCLC to be used in combination with gefitinib independently of the EGFR mutation profile. The intrapleural administration of ATO resulted in a reduction in the effusion volume, as well as the number of cells. ATO exerted antiproliferative activity in cancer cells independently of the EGFR mutations, apparently by disturbing the mTOR and PI3K pathways, in addition to inducing the autophagic process. These articles illustrate that drug combinations may enhance interference across a broader range of signaling pathways.

Time is crucial when we talk about cancer; for this reason, in this Special Issue, the repurposing of drugs was the focus of two papers. In Harich et al.’s study (Article 4), the Na^+^/K^+^ pump inhibitor ouabain was examined for its impact on various components of the tumor microenvironment, including cancer cells, tumor-associated fibroblasts (TAFs), and mesenchymal stem cells (MSCs). Ouabain demonstrated multiple effects, including pro-apoptotic activity, the reduction in VEGF-R2 and Her2 expression in SKBR3 cells, and a decrease in the markers of cell proliferation in TAF and MSC. The disruption in the expression of Na^+^/K^+^ pump subunits led to decreased adhesion to VCAM in all three cell types, positioning ouabain as a promising candidate for multiple microenvironment components. In Roddy et al.’s study (Article 5), they identified the regulation of the immune system as a crucial factor in glioblastoma recurrence (recGBM). Differentially expressed genes in recGBM, such as NFAT and MHC class II processing and presentation, are directly associated with immune system regulation. Their computational analysis of gene expression changes from initial GBM to recurrent GBM identified pathways altered in cancer, allowing for a correlation with FDA-approved drugs. This approach facilitates drug repurposing for reversing recGBM. The study highlights several drugs, including the diabetes medication rosiglitazone and the histamine H2-receptor antagonist nizatidine. Therefore, the papers from Harich and Roddy converge in the exploration of drug repositioning as an economical and time-saving strategy to broaden the array of available cancer therapeutic agents.

While drug repurposing presents a promising perspective in cancer therapy, the development and discovery of new active molecules and techniques remain central concerns in the scientific community. Consequently, the contributions in this Special Issue attended this pivotal topic. Abdelaal et al.’s research (Article 6) analyzed the potential of the iron chelator SK4 synthesized by their group. Using two cancer cells lines, SKOV3 and MDA-MB-231, proteomic analysis revealed disturbance in cell metabolism with the differential expression of 54 proteins from the energy metabolism pathway in common to both cell lines. Treated cells exhibited reduced basal and maximal respiration confirmed by metabolomic analysis, indicating decreased mitochondrial ATP production and an increase in the glycolysis intermediate glucose-1-phosphate. Presenting plant-derived products as good candidates as chemotherapeutical agents, Machado et al. (Article 7) demonstrated that dichloromethane partition from *Piper cernuum* leaves efficiently inhibits oral squamous cell carcinoma lines in vitro. Moreover, the partition significantly reduced the formation of pre-malignant lesions in animal models of the induction of oral cancer, improving the overall survival of mice and exerting low chronic toxicity. From the partition, unprecedented aporphine alkaloids in *P. cernuum* were isolated that were associated with the most selective and pro-apoptotic fractions of the tested partition. By taking advantage of the migratory profile of mesenchymal stem cells (MSCs), Quiroz-Reyes et al. (Article 8) transfected MSC with the transgenes, encoding the pro-regulator of antitumoral immune response IL-12 or the apoptosis inducer sTRAIL. In the in vivo model with balb-c mice with a solid tumor induced by the intramuscular application of L5178Y lymphoma cells, the intratumoral injection of transgenic MSC expressing IL-12 alone or in combination with sTRAIL resulted in reduced tumor growth and enhanced overall survival. However, this intervention did not block metastasis, despite a decrease in the migration of lymphoma cells.

Understanding the mechanisms implicated in acquired resistance benefit research, such as those mentioned previously, promotes the development of molecules with the potential to selectively inhibit or activate pathways that may induce cancer cells death. Articles published in this Special Issue highlight the mechanisms associated with cancer resistance, offering valuable insights for therapeutical targeting. The contribution by Lefebvre et al. (Article 9) demonstrated that triple-negative breast cancer cells resistant to Cabozantinib (c-Met inhibitor), and to the EGFR inhibitor Erlotinib, exhibited the overexpression of kinases AKT, SYK, and ERK and CK2, CDK1, and CDK7 after a comprehensive proteome, phosphoproteome, and kinome analysis. This overexpression led to significant alterations in invasion, migration, and proliferation rates compared to sensitive cells. The use of an AKT inhibitor in combination with Erlotinib in resistant cells reduced colony formation, confirming the involvement of AKT in the resistance mechanism. The screening pipeline in the work published by Shamloo et al. (Article 10) focused on the role of long non-coding RNA (lncRNAs) in BRAF inhibitor (Vemurafenib)-resistant melanoma cells. The researchers analyzed the expression profile of lncRNAs in resistant cells, followed by confirmation of their involvement in BRAFi resistance using the CRISPR activation method. Each lncRNA was overexpressed in parental cells, which were then treated with Vemurafenib to validate the BRAFi-resistant phenotype. The study revealed that the identified lncRNAs were directly related to cell survival and resistance, reinforcing the importance of investigating these regulatory molecules in the context of cancer. The study by Wu et al. (Article 11) performed extensive analysis using databases to develop a prognostic model for lung adenocarcinoma (LUAD). Their experimental findings confirmed that RNA-binding protein RBM10 expression in both cancer cells and surrounding cells is associated with low-risk LUAD. Therefore, RBM10 emerges as a potential prognostic marker for LUAD. The paper from Kang et al. (Article 12) presented evidence that osteosarcoma (OS) cells, regardless of their p53 expression status (normal or abnormal), were effectively inhibited by the RNA pol I inhibitor CX-5461 in a xenograft model involving immunosuppressed mice established using SJSA and 143B cell lines. Additionally, an allograft model in immunocompetent mice, utilizing K7M2 cells, demonstrated a reduction in tumor size. These results highlight the inhibition of RNA pol I as a good strategy for OS therapy. Twomey and Zhang (Article 13) contributed to the understanding of the transition of breast cancer (BC) cells into stem-like circulating phenotype. They demonstrated that BC cells cultured in suspension showed stemness marker CD44^+^/CD24^−^ as an indicator of this process, and a high expression of carbonic anhydrase IX (CAIX) was associated with the anoikis phenotype. The elucidated information holds potential significance for the detection of circulating tumor cells derived from breast cancer. The extensive range of targets explored in these works emphasizes the challenge of effectively targeting all the pathways, resulting in difficulties in chemotherapy.

The work from Wermelinger et al. (Article 14) underscores the importance of another target in cancer therapy: the p53 pathway. The biological activity of a new chalcone with great potential against oral, colon, and hepatocarcinoma by inducing apoptosis was attributed to the inhibition of the p53-regulatory enzyme MDM2. Encouragingly, the chalcone demonstrates promising in vivo results with low acute toxicity in mice.

Beyond mechanisms within cancer cells, other cells or structures favor carcinogenesis. Therefore, the involvement of the tumor microenvironment in the acquisition of cancer resistance was addressed in the article by Kusamura et al. (Article 15). It was characterized that, in Pseudomyxoma Peritonei (PMP), the immunosuppressive cytokine GM-CSF is overexpressed by tumor cells with both mutated and wild-type KRAS. Additionally, wild-type GNAS tumor cells showed the increased activation of the A2AR axis in comparison to mutated-GNAS cells. The expression of CD73/CD39, related to adenosine signaling, along with GM-CSF overexpression, was directly associated with the suppression of TCD8+ and NK cells, as well as the positive maturation of type M2 macrophages. These findings contribute to our comprehension of the importance of immune system regulation for cancer therapy.

## 3. Review Articles, Technical Note, and Commentary

As part of the proposal for this Special Issue, seven reviews were written about drugs that aim to avoid resistance to cancer treatment or new novel cancer-related targets. The paper written by Haroon et al. (Review 1) reviewed the role of para-aminobenzoic acid (PABA) as a commonly used building block in pharmaceuticals. They elucidate the chemical characteristics of PABA that characterize it as an important platform for the development of different molecules with various medical applications, such as anticancer, anti-cholinesterase, anti-microbial, anti-inflammatory, and antiviral. In addition, regarding the development of medications against drug resistance to cancer treatment or cancer prevention, the use of natural products such as polyphenols was addressed. The review from Aatif (Review 2) provided an overview of the study of nanocarriers aimed at enhancing the oral bioavailability of polyphenols. The review emphasizes the diverse anticancer effects exhibited by polyphenols, including pro-apoptotic, anti-angiogenic, cell cycle arrest, and metastasis inhibition across various types of cancer. Utilizing nanocarriers emerges as a strategic approach, facilitating the encapsulation, preservation, and distribution of these compounds. Complementarily with what was covered in the previous review, Farhan (Review 3) discussed the effectiveness of natural polyphenolic compounds against cancer drug resistance. The article explores acquired and intrinsic drug resistance mechanisms, emphasizing polyphenols’ potential to modulate drug resistance, act as chemopreventive agents, and enhance the efficacy of standard treatments. Polyphenols can contribute to overcoming drug resistance by modifying cellular metabolism, inhibiting glucose transport and ATP synthase function, as well as acting as an inhibitor of drug efflux transporters. They were also related to acting in DNA methylation or regulating microRNAs.

Moreover, novel targets for cancer therapy have also been presented. Chang’s review article (Review 4) detailed three E3 ubiquitin ligases: RNF126,168, and CUL1, highlighting their function in cell cycle regulation, DNA damage repair, cell cycle regulation, and cell death. This review reinforced those mutations, changes in expression levels, and other alterations of ubiquitination factors which are frequent in uncontrolled cell proliferation, a hallmark of cancer, by elucidating the ubiquitin pathway in cancer cell proliferation control as an interesting target for anticancer therapy and suggesting potential strategies for cancer therapy utilizing those E3 ligases. Equally important, Pintor-Romero et al. (Review 5) described the potential role of syntenin-1 as a novel biomarker and potential therapeutic target of its overexpression in several types of cancer. Syntenin-1 participates in the metastatic process in different types of cancer and chemoresistance in colorectal cancer. This amino acid protein has been suggested as a potential biomarker and therapeutic target in cancer, helping the effectiveness of prognostics and immunotherapies. Additionally, the use of antibodies has also been encompassed in cancer therapies. Shah et al. (Review 6) wrote a review of the bispecific antibody to target metastatic non-small-cell lung cancer, specifically the exon20ins mutation with the epidermal growth factor receptor. This review brings the pharmacology, pharmacokinetics, and pharmacodynamics of Amivantamab-Vmjw, as well as its clinical trial efficacy. Amivantamab-vmjw shows a benefit in the overall response rate of the early findings of the CHRYSALIS trial, and a relatively tolerable safety and toxicity profile. Amivantamab-vmjw is an FDA-approved antibody based on early efficacy data. It can be used as a monotherapy and in combination with other therapies in non-small-cell lung cancer harboring EGFR exon20ins mutations, and its cost and dose were explained in this review. Expanding on the topic of immunotherapy, Rodríguez-Nava et al. (Review 7) wrote a review describing the structure, function, and application of monoclonal antibodies and alternative formats in the diagnosis or treatment of cancer. They presented the historical use of monoclonal antibodies in different types of cancer, the development of novel antibodies targeting different epitopes, and the recent use of novel antibodies for tumor solid cell treatment. The application of conjugated antibodies and drugs was highlighted in the way of their specificity to target antigens to increase antitumoral activity due to their specificity and high affinity. The mechanism of action of the single-chain fragment variable (scFV) against tumor cells was also explained with their use combined with drugs, antibodies, or immune cells.

In addition to the research and review articles outlined, one technical note and a commentary have also been included to complement this Special Issue. The technical note by Chen et al. (Technical note 1) established a cost-effective modified inverse-PCR protocol for synthesizing site-directed nonsense mutations, facilitating the study of p53 mutations. This method proves valuable in investigating drug candidates capable of restoring p53 expression in the presence of nonsense mutations. And the commentary by Meriggi et al. raised a discussion about reconsidering the current dosing of immunotherapy (Commentary 1). According to their commentary, studies suggest that low doses of immunotherapy could yield comparable results to the current doses administered in patients. This could have a positive impact on health systems globally, reducing the cost of treatment and benefiting patients or countries with limited financial resources.

## 4. Take-Home Message

The articles in this Special Issue cover topics aimed at increasing knowledge around the cellular, molecular, and genetic pathways involved in drug resistance in cancer treatment and novel targets for cancer therapy. The text highlights the importance of understanding the cellular and molecular mechanisms that contribute to chemotherapy resistance and novel targets to fight cancer cells. The overall objective of this Special Issue is to provide a comprehensive overview of research areas that examine mechanisms and new approaches to cancer therapies.
